# Prevalence, Incidence, and Associated Factors of Possible Sarcopenia in Community-Dwelling Chinese Older Adults: A Population-Based Longitudinal Study

**DOI:** 10.3389/fmed.2021.769708

**Published:** 2022-01-10

**Authors:** Zi Chen, Mandy Ho, Pui Hing Chau

**Affiliations:** School of Nursing, The University of Hong Kong, Hong Kong, Hong Kong SAR, China

**Keywords:** prevalence, incidence, possible sarcopenia, risk factors, epidemiology

## Abstract

**Purpose:** This study aimed to assess the prevalence, incidence, and associated factors of possible sarcopenia in a nationwide representative sample of the community-dwelling older Chinese population.

**Methods:**This study used the data of participants aged 60 years and over from the China Health and Retirement Longitudinal Study (CHARLS). Data on participants from three waves (2011–2015) of CHARLS were extracted. Possible sarcopenia was defined as low muscle strength or low physical performance, based on the Asian Working Group for Sarcopenia 2019 consensus. We first described baseline prevalence and four-year incidence of possible sarcopenia. Then multiple logistic regression and multivariable parametric proportional hazard model with Weibull distribution were used to examine the association of risk factors with baseline prevalence and four-year incidence of possible sarcopenia, respectively.

**Results:**The prevalence of possible sarcopenia was 46.0%. The four-year incidence of possible sarcopenia was 11.9 per 100 person-years. Multivariable analysis revealed that advanced age and depressive symptoms were associated with increased prevalence of possible sarcopenia, while receiving education and moderate or high physical activity were associated with a lower risk of possible sarcopenia prevalence. For incidence, only advanced age was associated with an increased risk of possible sarcopenia incidence.

**Conclusion:**Our study revealed the substantial burden of possible sarcopenia and related risk factors in community-dwelling settings in China. It highlighted the importance of early detection and intervention in this subclinical group for the prevention of sarcopenia.

## Introduction

Advancing age is accompanied by a series of physiological changes in body composition, one of which is characterized as the gradual decrease in muscle quantity and quality (1). When low skeletal muscle mass coexisted with low muscle function (muscle strength or physical performance), this geriatric syndrome is termed sarcopenia (2). Previous evidence reported the prevalence of sarcopenia, defined by the Asian Working Group for Sarcopenia (AWGS) 2014, ranged from 5.5 to 25.7% among the Asian population (3). For Chinese community-dwelling older adults, the pooled prevalence was 11–14% (4, 5). In the eastern area of China, the prevalence rate was 9.7% (6). Sarcopenia is associated with a series of adverse health outcomes, such as falls, fractures, frailty, physical disability, and hospitalization (7). However, sarcopenia develops insidiously, even with no obvious symptom in the early stage. In general, people with sarcopenia are not aware of this disorder until progressively decreased muscle function becomes severe enough, such as the occurrence of physical dependence (8). Therefore, to enable timely intervention, early screening and identifying the vulnerable individuals who are on the way to sarcopenia before resulting in adverse outcomes should be at the forefront of sarcopenia management.

Measuring muscle mass is an indispensable procedure in the diagnosis of sarcopenia. However, assessing muscle mass is still a challenge in primary care settings where reliable and validated diagnostic equipment is not easily accessible. To promote early identification of people at risk of, or on the way to, sarcopenia and raise awareness of sarcopenia prevention in primary care settings, the AWGS 2019 consensus proposes a new concept named “possible sarcopenia,” which refers to poor muscle strength or low physical performance (3). According to the AWGS 2019 algorithm for sarcopenia, the SARC-F or SARC-CalF questionnaire could be used for case-finding in community settings (3). For those whose SARC-F ≥ 4 or SARC-CalF ≥ 11, muscle strength and physical performance should be assessed to detect whether the possible sarcopenia exists. If possible sarcopenia is identified, early lifestyle intervention and preventive service should be provided for this vulnerable group (3).

The epidemiological information of possible sarcopenia is limited. At present, three cross-sectional studies have reported the prevalence of possible sarcopenia based on the AWGS 2019 consensus (9–11). One was conducted in Singapore, which recruited 536 adults aged from 21 to 90 years, with a prevalence of 15.3% (10). The other study was conducted in Korea, which recruited 2,123 older adults, with the prevalence as 20.1% in men and 29.2% in women (9), and the third one studied 6,172 Chinese participants, with the prevalence as 38.5% (11). However, no study reported the incidence of possible sarcopenia using the population-based longitudinal data, only the incidence of sarcopenia was examined in previous research (12, 13). Furthermore, no study examined the risk factors of the incidence of possible sarcopenia.

The purpose of this study was to estimate the prevalence and incidence of possible sarcopenia, according to the definition of the AWGS 2019 consensus, and to examine potential risk factors for both using a nationwide representative sample of community-dwelling older Chinese population aged 60 years and above. As a newly proposed concept in the updated guideline for sarcopenia, possible sarcopenia is less investigated. Epidemiological evidence of possible sarcopenia, such as prevalence and incidence, is the first step for decision-making regarding resource allocation in healthcare (e.g., prevention, screening, and treatment) and to develop preventive routines or healthcare services tailored to the growing older population. Identifying risk factors of possible sarcopenia could further help to prioritize screening and prevention programs for the particular subgroup(s).

## Methods

### Design

This study was the secondary analysis of the China Health and Retirement Longitudinal Study (CHARLS).

### Data Sources and Participants

This study used data from CHARLS, which is an ongoing longitudinal survey targeting a nationally representative sample of Chinese adults aged 45 years and over. Details of CHARLS have been reported elsewhere (14). Briefly, the baseline survey of the CHARLS was conducted in 2011, which involved 17,708 respondents (response rate: 80.5%) from 28 provinces in China. These participants were followed up every 2 years from 2011 to 2015. In this study, we included data from participants aged 60 years and over at the first wave (2011). Following previous studies on sarcopenia, we excluded participants with psychiatric or cognitive disorders, or cancer (15–18), because these conditions might affect their response to the survey or induce more uninformative censoring during follow-up. We also excluded those who had missing data either in the handgrip strength test or five-time chair stand test at baseline, because possible sarcopenia was defined based on the results of these two tests (3). Ethics approval for the data collection in CHARLS was obtained by the original authors of CHARLS from the Biomedical Ethics Review Committee of Peking University (IRB00001052-11015).

### Measures

#### Possible Sarcopenia

Possible sarcopenia, at all three waves, was defined based on the AWGS 2019 consensus, as low muscle strength or low physical performance (3).

Muscle strength was assessed by handgrip strength. In CHARLS, handgrip strength was measured with the mechanical dynamometer (YuejianTM WL-1000, Nantong, China). Participants were instructed to bend the elbow with 90° and squeeze the dynamometer as hard as they can for a couple of seconds. For those unable to stand unassisted, sitting was allowed. In line with AWGS 2019 recommendation, each hand was tested twice separately, and the maximum reading of four measures was used to reflect handgrip strength. Low muscle strength was defined as the handgrip strength <28 kilograms (kg) in men and <18 kg in women (3). The handgrip strength would be coded as missing if participants did not appear to use full effort during the test, or if the measuring position was lying down or unknown, or if outlier data (defined as > 99 percentile or <1 percentile) were recorded (19, 20).

Physical performance was assessed by the five-time chair stand test. Participants were instructed to sit down and keep their arms folded across the chest. Then they were asked to stand up and sit down at their fastest pace five times consecutively, without stopping and moving arms. The time needed to finish the test was recorded by the examiner. As recommended by AWGS 2019, low physical performance was defined as needing 12 s or more to complete the task (3). Participants who tried but could not complete this test would be regarded as having low physical performance. Similarly, outlier data (defined as > 99 percentile or <1 percentile) of this test would be coded as missing (19).

#### Risk Factors for Possible Sarcopenia

Based on previous evidence on sarcopenia, similar risk factors were considered for possible sarcopenia, including age, gender, education level, marital status, residence, smoking and drinking status, physical activity (PA), depression, body mass index (BMI), and multimorbidity (10, 13).

Age was divided into three subgroups: 60–69, 70–79, and 80 and above. The highest education level was divided into four groups: illiterate, primary school, secondary school, and high school and above. The marital status of participants was categorized into two groups: married vs. single, divorced, or widowed. Rural or urban residence was determined based on the administrative division from the National Bureau of Statistics China (21).

Smoking and drinking status were grouped into three categories: never, ever but quit, and current use. As for PA level, the CHARLS collected information regarding the intensity, duration, and frequency of PA in a usual week. Three types of intensity (vigorous PA, moderate PA, and walking) and discrete time duration of PA were collected. In this study, we first calculated the volume of each type of PA by multiplying duration per day (minutes/day) with frequency (days), then transformed the volume into a metabolic equivalent value (MET) (walking = 3.3 MET, moderate PA = 4 MET, and vigorous PA = 8 MET) (22). Because CHARLS did not measure the exact duration of time per day regarding each type of PA, we could only obtain the range of duration time (0–30, 30–120, 120–240, and 240 min above). In that case, the daily duration of each type of PA was assessed by the average value of each time range. The total volume of PA (MET-minutes/week) was calculated as the sum of volumes of vigorous PA, moderate PA, and walking. Based on the IPAQ scoring protocol, the PA level was divided into three groups: low, moderate, and high PA level (22).

Depression was measured with the validated 10-item Center for Epidemiologic Studies Depression Scale short form (23, 24). Participants were asked to rate the frequency of each mood or symptom that occurred in the last week. Each item was scored ranging from 0 to 3. The total scores were calculated by summing all the item scores after reversing two items that were positively formulated (items 5 and 8). Depression was defined by the total score ≥12 (23).

Body mass index (BMI) was calculated as the weight in kilograms divided by the square of height in meters. BMI status was categorized into four groups: obese (BMI ≥ 25 kg/m^2^), overweight (23 kg/m^2^ ≤ BMI <25 kg/m^2^), normal (18.5 kg/m^2^ ≤ BMI <23 kg/m^2^), and underweight (BMI <18.5 kg/m^2^) (25). Multimorbidity refers to the coexisted presence of multiple chronic diseases (26). At present, the operational definition of multimorbidity in current literature varied a lot in the selection of different diseases and the cutoff point of the number of conditions (27). Based on a systematic review, the co-occurrence of two or more chronic conditions was most commonly used to define multimorbidity (27). Therefore, in this study, multimorbidity was defined as the existence of two or more chronic non-communicable diseases (28). The CHARLS investigated 14 diagnosed non-communicable diseases such as cardiovascular diseases, chronic lung diseases, liver diseases, and digestive diseases, etc. In this study, we only used 11 non-communicable diseases (psychiatric diseases, cognitive disorders, and cancer were excluded as these were the exclusion criteria of the participants) to define multimorbidity.

### Statistical Analysis

Descriptive statistics on the sample characteristics were calculated for the total analytical sample. For incidence assessment, we only analyzed the data from participants who were free of possible sarcopenia at baseline. Those who lacked data to identify possible sarcopenia at both two follow-up waves (2013 and 2015) were excluded. Furthermore, to restrict interval censoring to within 2 years, we also excluded those who only lacked data on possible sarcopenia at the second wave (2013). Incidence proportion was calculated as new cases during the follow-up divided by the total number of at-risk subjects being followed. The four-year incidence rate of possible sarcopenia was calculated as new cases during 2011–2015 divided by the person-years of follow-up.

For the identification of risk factors, we only analyzed data from participants without missing data in the potential risk factors at baseline. We used multivariable logistic regression to examine the adjusted association between risk factors and prevalent possible sarcopenia at baseline. Adjusted odds ratio (OR) was estimated with 95% CIs. For the incidence of possible sarcopenia, the onset time could not be exactly detected because the CHARLS collected data every 2 years. For new cases during the four-year follow-up, the onset time was only known to lie in an interval time between the last wave of free of possible sarcopenia and the wave of the new diagnosis of possible sarcopenia. Therefore, the onset time was regarded as interval censoring. For those free of possible sarcopenia until the third wave, the data were regarded as right censoring. Right censored data can be regarded as the special case of interval censoring, with the interval unbounded on the right (29). For interval-censored data, if the lower bound, midpoint, or upper bound of the interval is assumed as the onset time, it may result in biased estimates because the inherent uncertainty of the exact onset time is ignored (30, 31). Parametric proportional hazards models are suitable to accommodate interval-censored data (32). Therefore, we assumed that the onset time of possible sarcopenia followed a Weibull distribution. A multivariable parametric proportional hazard model with Weibull distribution was fitted to detect the adjusted association of risk factors with the four-year incidence of possible sarcopenia. Adjusted hazard ratio (HR) was estimated with 95% CIs. When performing the above multivariable models, the potential multicollinearity was checked. The cutoff of correlation coefficient <0.8 was considered acceptable (33).

Considering the multistage probability sampling design and non-response in the CHARLS data, individual sampling weights with non-response adjustment were taken into account in the analysis. All analyses were conducted using Stata15.0 (34). Significance was set at the 0.05 level, with the two-tailed test.

## Results

### Prevalence and Incidence of Possible Sarcopenia

**Figure 1:**
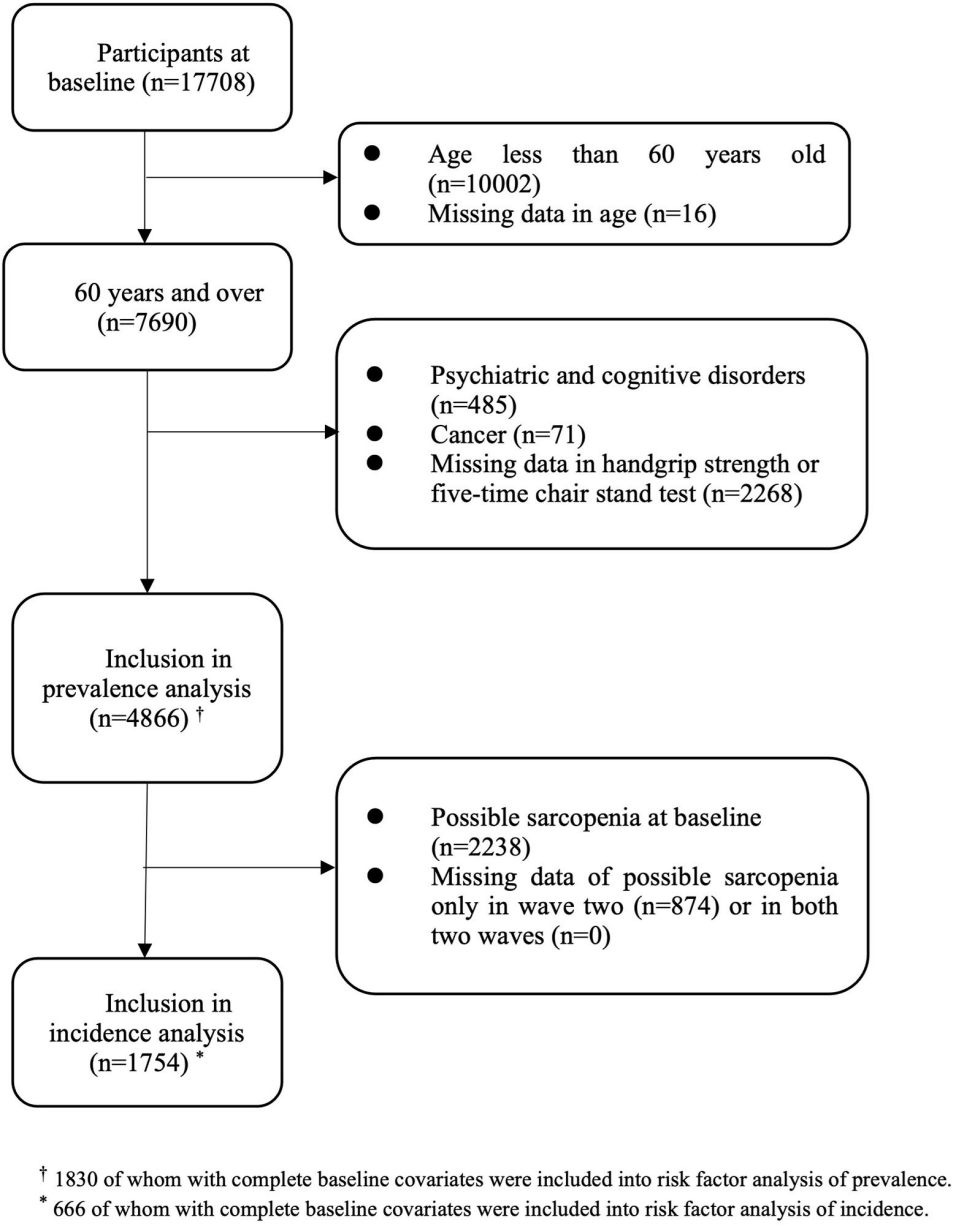
Flowchart of participants selection.

Among the 17,708 participants in the first wave, 7,690 were aged 60 years and over. After excluding those with psychiatric and cognitive disorders (*n* = 485), cancer (*n* = 71), and those with missing data either in handgrip strength or the five-time chair stand test (*n* = 2,268) at baseline, a total of 4,866 participants were eligible for inclusion in analyzing baseline prevalence (50.3% of men, 49.7% of women; mean age: 67.7 ± 6.4 years old) (Figure 1). A total of 2,238 participants had possible sarcopenia at baseline, giving an overall prevalence rate of 46.0% (95% CI: 44.6–47.4%). The gender-specific prevalence of possible sarcopenia was 40.8% (95% CI: 38.8–42.7%) for men and 51.3% (95% CI: 49.3–53.3%) for women. Of those with possible sarcopenia, 304 participants (13.6%) only had low muscle strength, 1,452 participants (64.9%) only had low physical performance, and 482 participants (21.5%) had both poor muscle strength and low physical performance. Sociodemographic characteristics of those included in the prevalence analysis were shown in Table 1.

**Table 1:** Characteristics of participants at baseline.

**Characteristics**	**All sample**		
	***N***	**Unweighted**	**Weighted**
**Gender (%)**			
Female	2,420	49.7	50.4
Male	2,446	50.3	49.6
**Age (%)**			
60–70	3,456	71.0	68.2
70–80	1,198	24.6	25.8
80 and above	212	4.4	6.0
**Education (%)**			
Illiteracy	1,763	36.2	35.2
Primary school	2,264	46.5	46.2
Secondary school	564	11.6	12.2
High school and above	275	5.7	6.4
**Marriage (%)**			
Married	3,862	79.4	76.7
Single, divorced or widowed	1,004	20.6	23.3
**Residence (%)**			
Urban	1,725	35.5	42.2
Rural	3,141	64.5	57.8
**Physical activity (%)**			
Low	431	21.5	23.4
Moderate	398	19.9	22.2
High	1,175	58.6	54.4
**Smoking status (%)**			
Never	2,782	57.5	58.7
Former	528	10.9	11.9
Current	1,531	31.6	29.4
**Drinking status (%)**			
Never	2,812	57.9	58.8
Former	554	11.4	10.9
Current	1,492	30.7	30.3
**CESD score, median (interquartile range, IQR)**		7.0 (9.0)	7.0 (8.0)
**Depression (%)**			
No	3,185	70.3	72.3
Yes	1,349	29.7	27.7
**BMI, median (IQR)**		22.4 (4.9)	22.6 (5.1)
**BMI category (%)**			
Underweight	454	9.7	9.6
Normal	2,165	46.1	43.9
Overweight	886	18.9	19.4
Obesity	1,186	25.3	27.1
**Numbers of chronic conditions, median**		1.0 (2.0)	1.0 (2.0)
**(IQR)**			
**Multimorbidity (%)**			
No	2,772	57.3	57.5
Yes	2,067	42.7	42.5
**Handgrip strength, median (IQR)**		29.2 (13.0)	29.0 (13.0)
**Chair stand test, median (IQR)**		10.8 (4.6)	10.9 (4.7)
**Muscle function (%)**			
Normal	2,628	54.0	53.0
Low strength only	304	6.3	6.2
Low physical performance only	1,452	29.8	30.3
Low strength and physical	482	9.9	10.5
performance			

For incidence analysis, we only included those free of possible sarcopenia at baseline (*n* = 2,628). After deleting those with insufficient data to identify possible sarcopenia at the second wave (*n* = 874), and at both two waves (*n* = 0), a total of 1,754 participants were included in the incidence analysis (Figure 1). At the end of wave three, there were 661 new cases of possible sarcopenia. The overall incidence proportion during the four-year follow-up was 37.7% (95% CI: 35.4–40.0%), with 36.9% (95% CI: 33.8–40.0%) for men and 38.7% (95% CI: 35.4–42.1%) for women. The four-year incidence rate of possible sarcopenia was 11.9 per 100 person-years (95% CI: 11.1–12.9). The gender-specific incidence rate was 11.7 per 100 person-years (95% CI: 10.5–13.0) for men and 12.3 per 100 person-years (95% CI: 11.0–13.7) for women.

### Risk Factors of Possible Sarcopenia

For risk factors analysis, we only included those free of missing data in baseline covariates (*n* = 1,830 for prevalence analysis and *n* = 666 for incidence analysis). Adjusted associations of risk factors with prevalent and incident possible sarcopenia were shown in Table 2. Participants who received education (primary: OR = 0.624, 95% CI: 0.479–0.812; secondary: OR = 0.558, 95% CI: 0.371–0.839; and high and above: OR = 0.250, 95% CI: 0.138–0.450, as compared to illiteracy) and had moderate or high PA level (moderate PA: OR = 0.622, 95% CI: 0.432–0.896 and high PA: OR = 0.501, 95% CI: 0.374–0.671, as compared to low PA level) were associated with lower prevalence of possible sarcopenia. However, those who were older (70–80 years: OR = 1.848, 95% CI: 1.390–2.458 and 80 years and above: OR = 3.737, 95% CI: 1.864–7.494, as compared to 60–70 years) and had depression symptoms (OR = 1.689, 95% CI: 1.316–2.167) presented an increased risk of prevalent possible sarcopenia. As for incident possible sarcopenia, only advanced age (70–80 years: OR = 1.516, 95% CI: 1.054–2.180; 80 years and above: HR = 4.035, 95% CI: 1.761–9.246, as compared with 60–70 years) was significantly associated with increased risk of incident possible sarcopenia.

**Table 2:** Risk factors of the prevalence and incidence of possible sarcopenia.

**Risk factors**	**Logistics regression model (*****n*** **=** **1,830)**				**Proportional hazard model (*****n*** **=** **666)**			
	**Adjusted OR**	**95% CI**		***P*-value**	**Adjusted HR**	**95% CI**		***P*-value**
		**Lower**	**Upper**			**Lower**	**Upper**	
**Gender**				0.189				0.554
Female	1				1			
Male	0.798	0.569	1.118		1.141	0.738	1.764	
**Age**				<0.001				<0.001
60–70	1				1			
70–80	1.848	1.390	2.458		1.516	1.054	2.180	
80 and above	3.737	1.864	7.494		4.035	1.761	9.246	
**Education**				<0.001				0.462
Illiteracy	1				1			
Primary school	0.624	0.479	0.812		0.818	0.590	1.136	
Secondary school	0.558	0.371	0.839		0.702	0.397	1.242	
High school and above	0.250	0.138	0.450		0.646	0.319	1.307	
**Marriage**				0.311				0.968
Married	1				1			
Single, divorced or widowed	1.161	0.870	1.550		0.991	0.641	1.532	
**Residence**				0.609				0.084
Urban	1				1			
Rural	1.069	0.828	1.380		1.346	0.960	1.887	
**Physical activity**				<0.001				0.619
Inactive	1				1			
Moderate	0.622	0.432	0.896		1.230	0.754	2.005	
High	0.501	0.374	0.671		1.030	0.686	1.546	
**Smoking status**				0.572				0.913
Never	1				1			
Ever but quit	1.192	0.766	1.856		1.007	0.534	1.897	
Current smoke	1.189	0.844	1.675		0.922	0.572	1.485	
**Drinking status**				0.351				0.658
Never	1				1			
Ever but quit	0.993	0.678	1.454		0.920	0.574	1.473	
Current drink	0.808	0.598	1.093		0.836	0.569	1.228	
**Depression**				<0.001				0.097
No	1				1			
Yes	1.689	1.316	2.167		1.305	0.953	1.788	
**BMI**				0.996				0.266
Underweight	1.015	0.668	1.542		1.560	1.003	2.426	
Normal	1				1			
Overweight	0.940	0.687	1.285		1.127	0.778	1.632	
Obesity	0.947	0.715	1.253		1.155	0.789	1.693	
**Multimorbidity**				0.287				0.356
No	1				1			
Yes	1.137	0.898	1.440		1.154	0.852	1.563	

## Discussion

This study examined the prevalence, incidence, and risk factors of possible sarcopenia in a nationwide representative sample of Chinese older adults. We found 46.0% of older adults had possible sarcopenia at baseline, which indicated that a large proportion of older adults had poor muscle function and were on the way to sarcopenia. Our estimated prevalence of possible sarcopenia was much higher than that in Singapore (15.3%) and Korea (20.1–29.2%) (9, 10). The cross-sectional investigation in Singapore recruited relatively younger adults, with 44.5% participants aged less than 60 years (age: 21–90 years and mean age: 58.5 years), which might account for the lower prevalence than our estimate (10). Compared with the other study in Korea, the difference in prevalence estimates was probably due to different operational definitions used to define possible sarcopenia (9). Furthermore, our prevalence from CHARLS 2011 was higher than that based on CHARLS 2015 dataset (46.0 vs. 38.5%) (11). The difference in the prevalence estimates might be due to the different lifestyles (e.g., alcohol drinking: 42.1 vs. 33.1% and smoking: 42.5 vs. 47.3%) between the two samples. Given that Wu and colleagues did not examine the PA level, it was unknown whether two samples also presented different PA levels.

At present, no other study examined the incidence rate of possible sarcopenia. Compared with existing evidence regarding the incidence of sarcopenia, the coexisted low muscle mass and low muscle strength/low physical performance, the incidence of possible sarcopenia reported in this study was much higher (12, 13, 35). For example, previous literature reported that the incidence proportion over a four-year period in community-dwelling older adults in China was 8.1% (35). Another cohort study found the three-year incidence rate of sarcopenia in British older adults was 3.7 per 100 person-years (13). The present study revealed the substantial burden of possible sarcopenia in community-dwelling settings in China, which indicated the high proportion of vulnerable older residents need early prevention of sarcopenia.

This study found the cross-sectional association of age, education, PA level, and depression with prevalent possible sarcopenia. The older age group showed a higher risk of prevalence, which indicated the advanced age was a significant independent risk factor of decreased muscle function. Consistent with existing evidence (10, 36), our findings indicated that older adults who received education and were physically active might be associated with better muscle function. Furthermore, our results showed the depressive symptom was associated with an increased risk of the prevalence of possible sarcopenia, which was consistent with current evidence regarding the cross-sectional association of depression with sarcopenia and its components, though different measurements were used to assess depression (37, 38).

In terms of incidence, only age was significantly associated with incident possible sarcopenia. Biological changes in tissues and organs during the aging process, such as the gradual decline in cellular metabolism and tissue regeneration, the decrease in muscle mass combined with a progressive increase in fat mass, and the function decline in the body system, are the pathogenic mechanism for the multisystem aging syndromes, such as frailty (39). Sarcopenia is regarded as the precursor syndrome or physical component of frailty (40). Though it is well established that the loss of muscle mass and function is accelerated with aging, the decline in muscle quantity and quality can be delayed or even reversed by timely lifestyle interventions involving exercise training and nutrition management targeting the older population (3). It is never too late for older adults to rebuild their muscles and preserve their function (41). Under this circumstance, the AWGS 2019 consensus proposes the entity of “possible sarcopenia” to promote our awareness of sarcopenia prevention in community and prevention settings. Our results showed no significant association between education level and the incidence of possible sarcopenia. Similar findings were also reported in previous cohort studies (12, 13). In view of the limited literature about possible sarcopenia, future longitudinal studies could consider further examining the predictive value of socioeconomic status on incident possible sarcopenia. Though PA is a well-known risk factor of sarcopenia, our finding showed no significant association between the PA level and the four-year incidence of possible sarcopenia. Similarly, the insignificant association of self-reported PA level and the incident sarcopenia was also reported in previous literature (12, 13). The insignificant effect of self-reported PA on the incident possible sarcopenia might reveal that PA had a relatively short-term effect; hence, the incidence of possible sarcopenia or sarcopenia was not associated with a baseline level of PA which had a time gap of several years. Future research could consider examining the longitudinal association between the trajectory of PA level and the incidence of possible sarcopenia or sarcopenia. Furthermore, previous evidence suggested that aerobic exercise had little effect on muscle strength or mass compared with resistance exercise (42). However, in this study, the PA only reflected the intensity level, and no information was available to further identify the type of exercise, such as aerobic or resistant exercise. Therefore, the PA level alone might be not enough to reveal the real association between PA and possible sarcopenia or sarcopenia. Moreover, our study also found an insignificant association between depression and the incidence of possible sarcopenia. As referred to the current evidence about the risk factors of incident sarcopenia, the insignificant association between depression and the incidence of sarcopenia was also reported in previous research (13, 43). Due to different definitions of depression and sarcopenia applied in current research, the results might be less comparable across studies. Future longitudinal studies are needed to further confirm the association between depression and the incidence of possible sarcopenia or sarcopenia using the same definition or diagnosis criteria to define depression, possible sarcopenia, or sarcopenia.

The major strength of this study was that we used a nationwide representative longitudinal database with large sample size. However, this study had some limitations. First, given that the CHARLS did not investigate the exact questions of the SARC-F questionnaire, we did not use the SARC-F or SARC-CalF questionnaire for case-finding. Instead, we directly assessed the muscle strength and physical performance to detect the possible sarcopenia. Future studies could consider constructing and validating SARC-F by using similar questions collected by the CHARLS, which could promote the sarcopenia assessment using nationwide population-based data. Second, in this study, we excluded participants with psychiatric, cognitive disorders, or cancer. However, due to data availability, we did not know the specific kinds and stages of psychiatric, cognitive disorders, and cancer. Therefore, we might exclude participants with just mild conditions that should be eligible in this study. Nevertheless, we conducted a supplementary analysis to include those with psychiatric, cognitive disorders, or cancer. The prevalence and four-year incidence of possible sarcopenia were 46.7% (95% CI: 43.4–48.1%) and 12.1 per 100 person-years (95% CI: 11.2–13.0), respectively. The estimates were similar to the main results. Furthermore, findings from the risk factor analysis were also consistent with our main results (Supplementary Table 1). Third, participants missing physical function tests at baseline might be frailer and more likely to suffer from possible sarcopenia. Therefore, this study might underestimate the prevalence of possible sarcopenia. Nevertheless, as those with possible sarcopenia at baseline had to be excluded from the analysis, the estimate of incidence of possible sarcopenia was less likely to be affected. Moreover, we did not include all possible risk factors of possible sarcopenia due to the lack of relevant data, such as dietary intake, nutritional status, osteoporosis, and the number of prescribed medications. Therefore, our results might be open to unmeasured confounders. Furthermore, some circulating biomarkers of sarcopenia, such as the C-reactive protein and interleukin 6, were not considered in this study. Although data about the C-reactive protein were available in the CHARLS dataset, we only focused on the risk factors of demographics and lifestyle behaviors in this study. It was because demographics and lifestyle factors were more accessible in community settings and more relevant to identify the at-risk population and inform the early lifestyle intervention. Future studies targeting the associations between biomarkers and possible sarcopenia could be conducted. Furthermore, the imprecise measurement of PA level might introduce uncertainty and might bias the estimation. Besides, there were missing values in some baseline covariates, by using the complete cases in the analysis, the statistical power might be reduced. However, we conducted a supplementary analysis with multiple imputations, and the results showed the factors identified from the complete data analysis remained significant (Supplementary Table 2). Finally, some baseline covariates such as PA level, smoking and drinking status, depression symptoms, and BMI might change during the four-year follow-up. However, we only examined the predictive role of the baseline level for all covariates on the four-year incidence. Future studies may investigate the association between changes in these variables and possible sarcopenia incidence.

## Conclusion

This study examined the prevalence and incidence of possible sarcopenia as well as the associated factors in Chinese community-dwelling older adults. Advanced age, not received education, physical inactivity, and depression symptoms were associated with an increased risk of possible sarcopenia prevalence, while only advanced age was associated with an increased incidence rate of possible sarcopenia. Early screening and lifestyle intervention for these at-risk populations are encouraged in the primary care service of sarcopenia prevention.

## Data Availability Statement

Publicly available datasets were analyzed in this study. This data can be found here: http://charls.pku.edu.cn/.

## Ethics Statement

The studies involving human participants were reviewed and approved by Biomedical Ethics Review Committee of Peking University. The patients/participants provided their written informed consent to participate in this study.

## Author Contributions

PC, ZC, and MH: study concept and design and critical revision of the manuscript for important intellectual content. ZC: data extraction and drafting of the manuscript. ZC and PC: analysis and interpretation of data. All authors contributed to the article and approved the submitted version.

## Conflict of Interest

The authors declare that the research was conducted in the absence of any commercial or financial relationships that could be construed as a potential conflict of interest.

## Publisher's Note

All claims expressed in this article are solely those of the authors and do not necessarily represent those of their affiliated organizations, or those of the publisher, the editors and the reviewers. Any product that may be evaluated in this article, or claim that may be made by its manufacturer, is not guaranteed or endorsed by the publisher.

## References

[1] Roubenoff R Castaneda C. Sarcopenia—understanding the dynamics of aging muscle. JAMA. (2001) 286:1230–1. 10.1001/jama.286.10.1230 11559270

[2] Santilli V Bernetti A Mangone M Paoloni M. Clinical definition of sarcopenia. Clin Cases Miner Bone Metab. (2014) 11:177–80. 10.11138/ccmbm/2014.11.3.177 25568649 PMC4269139

[3] Chen L-K Woo J Assantachai P Auyeung T-W Chou M-Y Iijima K. Asian working group for Sarcopenia: 2019 consensus update on sarcopenia diagnosis and treatment. J Am Med Dir Assoc. (2020) 21:300–7. 10.1016/j.jamda.2019.12.012 32033882

[4] Xin C Sun X Lu L Shan L. Prevalence of sarcopenia in older Chinese adults: a systematic review and meta-analysis. BMJ Open. (2021) 11:e041879. 10.1136/bmjopen-2020-041879 34413096 PMC8378367

[5] Tian S Xu Y Han F. Prevalence of Sarcopenia in the community-dwelling, elderly Chinese population: a systematic review and meta-analysis. Lancet. (2017) 390:35. 10.1016/S0140-6736(17)33173-2

[6] Huang J He F Gu X Chen S Tong Z Zhong S. Estimation of sarcopenia prevalence in individuals at different ages from Zheijang province in China. Aging. (2021) 13:6066–75. 10.18632/aging.202567 33601336 PMC7950223

[7] Woo J Leung J Morley J. Defining sarcopenia in terms of incident adverse outcomes. J Am Med Dir Assoc. (2015) 16:247–52. 10.1016/j.jamda.2014.11.013 25548028

[8] Visvanathan R Chapman I. Preventing sarcopaenia in older people. Maturitas. (2010) 66:383–8. 10.1016/j.maturitas.2010.03.020 20413231

[9] Kim M Won CW. Sarcopenia in Korean community-dwelling adults aged 70 years and older: application of screening and diagnostic tools from the Asian working group for Sarcopenia 2019 update. J Am Med Dir Assoc. (2020) 21:752–8. 10.1016/j.jamda.2020.03.018 32386844

[10] Pang BWJ Wee S-L Lau LK Jabbar KA Seah WT Ng DHM. Prevalence and associated factors of sarcopenia in singaporean adults—the yishun study. J Am Med Dir Assoc. (2021) 22:885.e1–e10. 10.21203/rs.3.rs-28685/v1 32693999

[11] Wu X Li X Xu M Zhang Z He L Li Y. Sarcopenia prevalence and associated factors among older Chinese population: findings from the China health and retirement longitudinal study. PLoS ONE. (2021) 16:e0247617. 10.1371/journal.pone.0247617 33661964 PMC7932529

[12] Yu R Wong M Leung J Lee J Auyeung TW Woo J. Incidence, reversibility, risk factors and the protective effect of high body mass index against sarcopenia in community-dwelling older Chinese adults. Geriatr Gerontol Int. (2014) 14 Suppl 1:15–28. 10.1111/ggi.12220 24450557

[13] Dodds RM Granic A Davies K Kirkwood TB Jagger C Sayer AA. Prevalence and incidence of sarcopenia in the very old: findings from the Newcastle 85+ Study. J Cachexia Sarcopenia Muscle. (2017) 8:229–37. 10.1002/jcsm.12157 27897431 PMC5377385

[14] Zhao Y Hu Y Smith JP Strauss J Yang G. Cohort profile: the China health and retirement longitudinal study (CHARLS). Int J Epidemiol. (2014) 43:61–8. 10.1093/ije/dys203 23243115 PMC3937970

[15] ChenQHaoQDingYDongB. The association between Sarcopenia and prealbumin levels among elderly Chinese inpatients. J Nutr Health Aging. (2019) 23:122–7. 10.1007/s12603-018-1130-530697620

[16] Hai S Wang H Cao L Liu P Zhou J Yang Y. Association between sarcopenia with lifestyle and family function among community-dwelling Chinese aged 60 years and older. BMC Geriatr. (2017) 17:187. 10.1186/s12877-017-0587-0 28821239 PMC5563006

[17] Lee WJ Liu LK Peng LN Lin MH Chen LK. Comparisons of sarcopenia defined by IWGS and EWGSOP criteria among older people: results from the I-Lan longitudinal aging study. J Am Med Dir Assoc. (2013) 14:528. 10.1016/j.jamda.2013.03.019 23664768

[18] Xu WH Chen T Shan Q Hu B Zhao M Deng XL. Sarcopenia is associated with cognitive decline and falls but not hospitalization in community-dwelling oldest old in China: a cross-sectional study. Med Sci Monit. (2020) 26:e919894. 10.12659/MSM.919894 31980594 PMC6998786

[19] Cai X Qiu S Liu S Lu Y Luo D Li R. Body-weight fluctuation and risk of diabetes in older adults: the China health and retirement longitudinal study (CHARLS). Diabetes Res Clin Pract. (2020) 169:108419. 10.1016/j.diabres.2020.108419 32891690

[20] Wu C Smit E Xue QL Odden MC. Prevalence and correlates of frailty among community-dwelling chinese older adults: the China health and retirement longitudinal study. J Gerontol A Biol Sci Med Sci. (2017) 73:102–8. 10.1093/gerona/glx098 28525586 PMC5861883

[21] National Bureau of Statistics China. Urban-Rural Division Codes For Statistics in 2011. Available online at: http://www.stats.gov.cn/tjsj/tjbz/tjyqhdmhcxhfdm/2011/index.html (accessed September 4, 2011).

[22] The IPAQ group. IPAQ Scoring Protocol. Available online at: https://sites.google.com/site/theipaq/scoring-protocol (accessed February 15, 2005).

[23] Radloff LS. The CES-D scale: a self-report depression scale for research in the general population. Appl Psychol Meas. (1977) 1:385–401. 10.1177/01466216770010030623302475

[24] Chen H Mui AC. Factorial validity of the center for epidemiologic studies depression scale short form in older population in China. Int Psychogeriatr. (2014) 26:49–57. 10.1017/S1041610213001701 24125553

[25] World Health Organization. Regional Office for the Western P. The Asia-Pacific perspective: Redefining Obesity and Its Treatment. Sydney, NSW: Health Communications Australia (2000).

[26] Calderón-Larrañaga A Vetrano DL Onder G Gimeno-Feliu LA Coscollar-Santaliestra C Carfí A. Assessing and measuring chronic multimorbidity in the older population: a proposal for its operationalization. J Gerontol A Biol Sci Med Sci. (2017) 72:1417–23. 10.1093/gerona/glw233 28003375 PMC5861938

[27] Johnston MC Crilly M Black C Prescott GJ Mercer SW. Defining and measuring multimorbidity: a systematic review of systematic reviews. Eur J Public Health. (2019) 29:182–9. 10.1093/eurpub/cky098 29878097

[28] Barnett K Mercer SW Norbury M Watt G Wyke S Guthrie B. Epidemiology of multimorbidity and implications for health care, research, and medical education: a cross-sectional study. Lancet. (2012) 380:37–43. 10.1016/S0140-6736(12)60240-2 22579043

[29] Chen D-G Sun J Peace KE. Interval-Censored Time-To-Event Data: Methods And Applications. Boca Raton, FL: CRC Press (2013). 10.1201/b12290

[30] Radke BR. A demonstration of interval-censored survival analysis. Prev Vet Med. (2003) 59:241–56. 10.1016/S0167-5877(03)00103-X 12835007

[31] Law CG Brookmeyer R. Effects of mid-point imputation on the analysis of doubly censored data. Stat Med. (1992) 11:1569–78. 10.1002/sim.4780111204 1439361

[32] Sparling YH Younes N Lachin JM Bautista OM. Parametric survival models for interval-censored data with time-dependent covariates. Biostatistics. (2006) 7:599–614. 10.1093/biostatistics/kxj028 16597670

[33] Mathias Harrer Pim Cuijpers Toshi A Furukawa Ebert DD. Doing Meta-Analysis in R: A Hands-on Guide 2019. Available online at: https://journaldown.org/MathiasHarrer/Doing_Meta_Analysis_in_R/ (accessed May 31, 2021).

[34] StataCorp. Stata Statistical Software: Release 15. College Station, TX: StataCorp LP (2017).

[35] YuXHouLGuoJWangYHanPFuL. Combined effect of osteoporosis and poor dynamic balance on the incidence of sarcopenia in elderly Chinese community suburban-dwelling individuals. J Nutr Health Aging. (2020) 24:71–7. 10.1007/s12603-019-1295-631886811

[36] Volpato S Bianchi L Cherubini A Landi F Maggio M Savino E. Prevalence and clinical correlates of sarcopenia in community-dwelling older people: application of the EWGSOP definition and diagnostic algorithm. J Gerontol A Biol Sci Med Sci. (2014) 69:438–46. 10.1093/gerona/glt149 24085400 PMC3968828

[37] Wang H Hai S Liu Y Cao L Liu Y Liu P. Association between depressive symptoms and sarcopenia in older Chinese community-dwelling individuals. Clin Interv Aging. (2018) 13:1605–11. 10.2147/CIA.S173146 30233157 PMC6130547

[38] Szlejf C Suemoto CK Brunoni AR Viana MC Moreno AB Matos SMA. Depression is associated with Sarcopenia due to low muscle strength: results from the ELSA-Brasil study. J Am Med Dir Assoc. (2019) 20:1641–6. 10.1016/j.jamda.2018.09.020 30409492

[39] Thillainadesan J Scott IA Le Couteur DG. Frailty, a multisystem ageing syndrome. Age Ageing. (2020) 49:758–63. 10.1093/ageing/afaa112 32542377

[40] Wilson D Jackson T Sapey E Lord JM. Frailty and sarcopenia: the potential role of an aged immune system. Ageing Res Rev. (2017) 36:1–10. 10.1016/j.arr.2017.01.006 28223244

[41] Cruz-Jentoft AJ Sayer AA. Sarcopenia. Lancet. (2019) 393:2636–46. 10.1016/S0140-6736(19)31138-9 31171417

[42] Takeshima N Rogers ME Islam MM Yamauchi T Watanabe E Okada A. Effect of concurrent aerobic and resistance circuit exercise training on fitness in older adults. Eur J Appl Physiol. (2004) 93:173–82. 10.1007/s00421-004-1193-3 15293053

[43] Yang L Smith L Hamer M. Gender-specific risk factors for incident sarcopenia: 8-year follow-up of the English longitudinal study of ageing. J Epidemiol Community Health. (2019) 73:86–8. 10.1136/jech-2018-211258 30368480

