# Supplementary material for: Prevalence, Incidence, and Associated Factors of Possible Sarcopenia in Community-Dwelling Chinese Older Adults: A Population-Based Longitudinal Study
Source: Front Med (Lausanne). 2022 Jan 10;8:769708. doi: 10.3389/fmed.2021.769708 (PMC8784542; doi:10.3389/fmed.2021.769708)
Supplement: Supplementary file 1 [file Data_Sheet_4.pdf]

Supplementary materials

Supplementary Table 1 Risk factors of the prevalence and incidence of possible sarcopenia (including those with cognitive, psychiatric disorders or cancer)

Risk factors	Logistics regression model (n=1943)				Proportional hazard model (n=695)			
	Adjusted OR	95% CI		*P*-value	Adjusted HR	95% CI		*P*-value
		Lower	Upper			Lower	Upper	
**Gender**				0.219				0.623
Female	1				1			
Male	0.816	0.590	1.129		1.113	0.726	1.706	
**Age**				<0.001				<0.001
60-70	1				1			
70-80	**1.746**	1.327	2.298		**1.586**	1.121	2.246	
80 and above	**3.725**	1.860	7.462		**4.185**	1.777	9.858	
**Education**				<0.001				0.465
Illiteracy	1				1			
Primary school	**0.604**	0.468	0.780		0.830	0.600	1.147	
Secondary school	**0.554**	0.375	0.818		0.760	0.440	1.313	
High school and above	**0.280**	0.162	0.483		0.607	0.300	1.231	
**Marriage**				0.348				0.499
Married	1				1			
Single, divorced or widowed	1.145	0.863	1.520		0.859	0.553	1.334	
**Residence**				0.634				0.151
Urban	1				1			
Rural	1.061	0.830	1.357		1.267	0.917	1.750	
**Physical activity**				<0.001				0.607
Inactive	1				1			
Moderate	**0.607**	0.427	0.864		1.215	0.751	1.964	
High	**0.466**	0.351	0.618		1.011	0.680	1.504	
**Smoking status**				0.667				0.862
Never	1				1			
Ever but quit	1.111	0.726	1.702		1.066	0.578	1.964	
Current smoke	1.161	0.837	1.611		0.933	0.587	1.482	
**Drinking status**				0.434				0.501
Never	1				1			
Ever but quit	0.992	0.688	1.432		0.806	0.503	1.289	
Current drink	0.833	0.622	1.114		0.819	0.566	1.184	
**Depression**				<0.001				0.036
No	1				1			

Yes	**1.696**	1.336	2.155		**1.381**	1.022	1.866	
**BMI**				0.966				0.251
Underweight	0.987	0.660	1.478		1.537	1.006	2.348	
Normal	1				1			
Overweight	0.960	0.707	1.304		1.047	0.722	1.519	
Obesity	0.933	0.712	1.224		1.105	0.757	1.615	
**Multimorbidity**				0.292				0.261
No	1				1			
Yes	1.131	0.900	1.421		1.186	0.881	1.598	

Supplementary Table 2 Risk factors of the prevalence and incidence of possible sarcopenia using imputed dataset

Risk factors	Logistics regression model (n=4866)				Proportional hazard model (n=1754)			
	Adjusted OR	95% CI		*P*-value	Adjusted HR	95% CI		*P*-value
		Lower	Upper			Lower	Upper	
**Gender**				0.467				0.047
Female	1				1			
Male	1.086	0.869	1.356		**1.303**	1.003	1.691	
**Age**				<0.001				<0.001
60-70	1				1			
70-80	**2.133**	1.754	2.593		**1.808**	1.433	2.282	
80 and above	**4.457**	2.838	6.998		**3.712**	2.148	6.414	
**Education**				<0.001				0.002
Illiteracy	1				1			
Primary school	**0.663**	0.556	0.789		**0.719**	0.579	0.892	
Secondary school	**0.527**	0.403	0.687		**0.585**	0.410	0.837	
High school and above	**0.245**	0.166	0.361		**0.520**	0.310	0.871	
**Marriage**				0.273				0.127
Married	1				1			
Single, divorced or widowed	1.113	0.919	1.349		1.229	0.943	1.601	
**Residence**				0.073				0.034
Urban	1				1			
Rural	1.173	0.985	1.397		**1.279**	1.018	1.607	
**Physical activity**				<0.001				0.806
Inactive	1				1			
Moderate	0.806	0.584	1.111		1.081	0.677	1.725	
High	**0.576**	0.424	0.781		0.956	0.630	1.451	
**Smoking status**				0.348				0.102
Never	1				1			
Ever but quit	0.846	0.602	1.189		0.799	0.564	1.132	
Current smoke	0.853	0.683	1.065		0.757	0.584	0.981	
**Drinking status**				0.008				0.481
Never	1				1			
Ever but quit	1.018	0.804	1.288		0.938	0.691	1.274	
Current drink	**0.739**	0.604	0.904		0.867	0.687	1.094	
**Depression**				<0.001				0.043
No	1				1			
Yes	**1.550**	1.296	1.855		**1.278**	1.007	1.622	

**BMI**				0.236				0.091
Underweight	1.196	0.925	1.547		1.373	1.027	1.835	
Normal	1				1			
Overweight	0.879	0.721	1.072		0.895	0.700	1.044	
Obesity	0.918	0.706	1.364		1.087	0.748	1.578	
**Multimorbidity**				0.372				0.165
No	1				1			
Yes	1.074	0.917	1.260		1.149	0.945	1.398	
